# Preservation of *Helicobacter pylori* CagA Translocation and Host Cell Proinflammatory Responses in the Face of CagL Hypervariability at Amino Acid Residues 58/59

**DOI:** 10.1371/journal.pone.0133531

**Published:** 2015-07-21

**Authors:** Mona Tafreshi, Nicolas Zwickel, Rebecca Jane Gorrell, Terry Kwok

**Affiliations:** 1 Department of Biochemistry and Molecular Biology, Monash University, Clayton, Victoria, Australia; 2 Department of Microbiology, Monash University, Clayton, Victoria, Australia; Indian Institute of Science, INDIA

## Abstract

Carriage of the CagA oncoprotein by the human gastric cancer-associated pathogen *Helicobacter pylori* is significantly associated with this typically benign chronic infection advancing to a potentially fatal outcome. However it remains to be elucidated why only a small subset of individuals infected with *H*. *pylori* CagA-positive strains develops gastric cancer. *H*. *pylori* translocates CagA into host cells using a type IV secretion apparatus that interacts with host integrin receptors via a three-amino-acid-residue RGD motif on the *H*. *pylori* protein CagL. The RGD motif of CagL also plays a major role in the induction of proinflammatory responses. Upstream of this motif is a conserved glycine flanked by four hypervariable amino acid residues (residues 58, 59, 61 and 62). Certain amino acid polymorphisms at 58 and 59 are significantly prevalent in strains from gastric cancer patients in particular geographic regions; Y58E59 is seen in Taiwan and D58K59 in India. In light of the seemingly contradictory findings of recent CagL mutagenesis studies, we have examined the contribution of sequence promiscuity specifically at CagL residues 58 and 59 to CagA translocation and *H*. *pylori*-mediated proinflammatory responses of gastric epithelial cells. Using isogenic mutants of *H*. *pylori* strains P12 and 26695 with amino acid substitutions at CagL residues 58 and 59, we determined that carriage of the polymorphisms Y58E59, D58K59, D58E59, N58E59 or N58K59 did not significantly alter the capacity of *H*. *pylori* to translocate CagA into, or induce IL-8 secretion in, host cells. Our findings, together with other recently published data, suggest that the variation at CagL residues 58 and 59 does not influence type IV secretion system function in isolation, but rather may work in concert with particular polymorphisms elsewhere in CagL to modulate disease progression.

## Introduction

Translocation of the bacterial oncogenic protein CagA into host gastric epithelial cells is an important pathogenic determinant of *Helicobacter pylori*. A number of bacterial and host cell factors are critical for CagA translocation, paramount among them being the interaction between the *H*. *pylori* protein CagL and host cell receptor integrin α_5_ß_1_ [1]. CagL is proposed to be expressed on the pilus of the *H*. *pylori* type IV secretion apparatus and is essential for CagA translocation into host cells and transformation of normal host cell phenotype into the so-called hummingbird elongation phenotype [1]. CagL also triggers host cell spreading [2] and suppresses expression of gastric H,K-ATPase subunit via upregulation of ADAM17 [3]. It is encoded by the 40-kb genetic locus, the *cag* pathogenicity island (*cag* PAI), which encodes also CagA and other components of the type IV secretion system responsible for CagA translocation [4].

Recent examinations of CagL have focused on solving its three-dimensional structure and investigating its contribution to disease progression. Analysis of the crystal structure of CagL suggests an elongated 4-helix bundle (α1, α2, α5 and α6) with 2 short perpendicular helices (α3 and α4) [5]. CagL also has the capacity to form a domain-swap dimer under non-physiological conditions [6] that may be suggestive of alternate structure(s) in differing biological contexts, such as following binding of interaction partners. The Arginine-Glycine-Aspartic Acid (RGD) motif at residues 76 to 78 of CagL, which is critical for integrin binding, is located in the center of helix α2. The flexibility of helix α2 is essential for RGD-dependent cell attachment [6]. Notably, the disordered and unresolved flexible hinge region between helices α1 and α2 contains a hypervariable amino acid sequence at residues 58 to 62, certain polymorphisms within which have been reported to correlate with severe disease progression in a geographically-dependent manner. Yeh et al first identified that *H*. *pylori* clinical isolates bearing the CagL amino acid polymorphism Y58E59 were significantly over-represented in Taiwanese gastric cancer patients compared to non-cancer patients [7]. In contrast, increased gastric cancer risk has subsequently been reported to associate with the CagL polymorphisms D58K59 in Indian patients [8] and N58 in a Mexican patient cohort [9]. Despite such geographical disparity, understanding the molecular basis for the association of CagL polymorphisms with gastric cancer risk is of great medical interest as it could provide crucial insights into the molecular mechanisms of *H*. *pylori*-induced gastric carcinogenesis.

*In vitro* attempts to clarify the mechanisms behind an association of Y58E59 with *H*. *pylori* pathogenesis have so far yielded contradictory results. A follow-up study by Yeh et al showed that substitution of Y58E59 with D58K59 in CagL significantly reduces CagA translocation and IL-8 induction, thus suggesting that the polymorphism Y58E59 contributes to enhanced *H*. *pylori* virulence [10]. In contrast, more recent work has suggested that a N58E59 to Y58E59 substitution in the CagL of *H*. *pylori* 26695 totally ablates CagL function in CagA translocation [11]. Whilst appearing to be contradictory, neither of these studies actually allowed definitive conclusions to be drawn about a specific role for amino acids 58 and 59 in CagL function and *H*. *pylori* pathogenesis: Tegtmeyer et al substituted amino acids at positions 60 and 62 in addition to a N58Y substitution; the mutagenesis approach and mutant characterization described by Yeh et al did not rule out significant polar effects in their substitution mutants [10].

Given these contradictory findings, we have specifically investigated the contribution of variation at CagL residues 58/59 alone to *H*. *pylori* pathogenesis by examining in detail the proinflammatory capacity and type IV secretion capability of *H*. *pylori* isogenic variants with amino acid substitutions at CagL residues 58 and/or 59.

## Materials and Methods

### Mammalian cell and bacterial culture

Mammalian cell and *H*. *pylori* culture was performed as described previously [12]. Gastric adenocarcinoma epithelial cell line AGS [13] was routinely cultured in RPMI (Invitrogen Corp, Carlsbad, CA, USA) supplemented with 10% (v/v) foetal bovine serum (FBS, Invitrogen) in a humidified incubator at 37°C with 5% CO_2_. *H*. *pylori* strains (S1 Table) were routinely cultured on GC agar (Oxoid, Basingstoke, UK) supplemented with 10% (v/v) horse serum (Invitrogen), vitamin mix, vancomycin and nystatin as described previously [14]. For cell-culture inoculum, *H*. *pylori* strains were cultured in brain heart infusion (BHI) or heart infusion (HI) broth (Oxoid) supplemented with 10% (v/v) FBS, vitamin mix, and vancomycin (Sigma, St Louis, MO, USA). *H*. *pylori* growth medium was further supplemented with chloramphenicol (Cm; 8 μg/ml for transformant selection, 4 μg/ml for routine culture) or kanamycin sulphate (Km;15 μg/ml) as required. All *H*. *pylori* culture was performed at 37°C under microaerobic conditions generated using the CampyGen system (Oxoid); broth cultures were shaken at 120 rpm. *E*. *coli* strain DH5α was propagated as described previously [15].

### *H*. *pylori* stimulation of AGS cells

AGS cells seeded in 24-well plates (5 x 10^4^ cells/well) were used 48 hours after seeding and maintenance media was replaced with 0.5 ml fresh media immediately prior to inoculation. Plate-grown *H*. *pylori* was used to inoculate HI broth to 0.1–0.2 O.D._550nm_ and grown overnight to 0.6–0.8 O.D._550nm_. AGS cell media was directly inoculated with *H*. *pylori* strains at a multiplicity of infection (moi) of 100 CFU/cell, as estimated by comparison of optical density against a reference standard curve. Spent culture media and cell lysates were harvested at 6 and 24 hours post-inoculation (hpi): spent media was analysed for IL-8 secretion using the human IL-8 ELISA set (BD Biosciences) and by immunoblot analysis of cell lysates for phosphorylated CagA using PY99 mouse monoclonal phosphotyrosine- and rabbit polyclonal CagA-specific antibodies (Santa Cruz Biotechnology) as previously described [12]. PY99-specific signal was detected using Amersham ECL Prime chemiluminescent substrate (GE Healthcare) and CagA-specific signal was detected using LumiGLO chemiluminescent substrate (KPL).

### *H*. *pylori* mutagenesis

A 26695 *cagL*-deletion mutant was generated as described previously for P12∆*cagL* [12]. To introduce amino acid substitutions at residues 58 and/or 59, a previously generated *cagL* knock-in construct, p26695*cagL*::*cat* was modified by inverse PCR using primer R-CagL58/59 together with the primers F-CagLN58/K59, F-CagLD58/E59, F-CagLY58/E59 or F-CagLD58/K59 (S2 Table.). Amplicons were phosphorylated, blunt-end ligated and used to transform *E*. *coli* DH5α. Substitutions were confirmed by sequencing of plasmid DNA. Knock-in of the *cagL* variants into P12∆*cagL* and 26695∆*cagL* was performed as described previously [12] and validated by fully sequencing an amplicon generated using primer pair HpcagNF/HpcagLR [12] specific for regions outside of the donor DNA sequence (chromatograms of mutated regions shown in S1 Fig).

### Reverse-transcription (RT)-PCR

Expression of *cagL* in AGS cell co-cultures was confirmed by *cagL*-specific reverse-transcription (RT)-PCR. Briefly, bacterial RNA was preserved in cells at 3 hpi by removing 0.3 ml culture media and adding 0.4 ml (2 vols) Bacterial RNA Protect reagent (Qiagen). After 5 mins at room temperature and centrifugation (10 mins, 5000 ×*g*), RNA was extracted from the pelleted bacterial cells using the RNA Isolate II kit (Bioline) according to manufacturer’s protocol for Gram negative bacteria. RNA (300 ng) was converted to cDNA using iScript Select cDNA synthesis kit (Bio-Rad) with the supplied random hexamer primers and PCR amplified using the *cagL*-specific primer pair cagLseq2/cagLseq3 [12] to produce a 320-bp product.

### Statistical analysis

Statistical analyses were performed using GraphPad Prism v6.0e (GraphPad Software, San Diego, USA). Significance (P value) was determined as specified in the text and/or figure legends using Fishers Exact test or one-way ANOVA with post-tests where appropriate.

## Results

**Fig 1:**
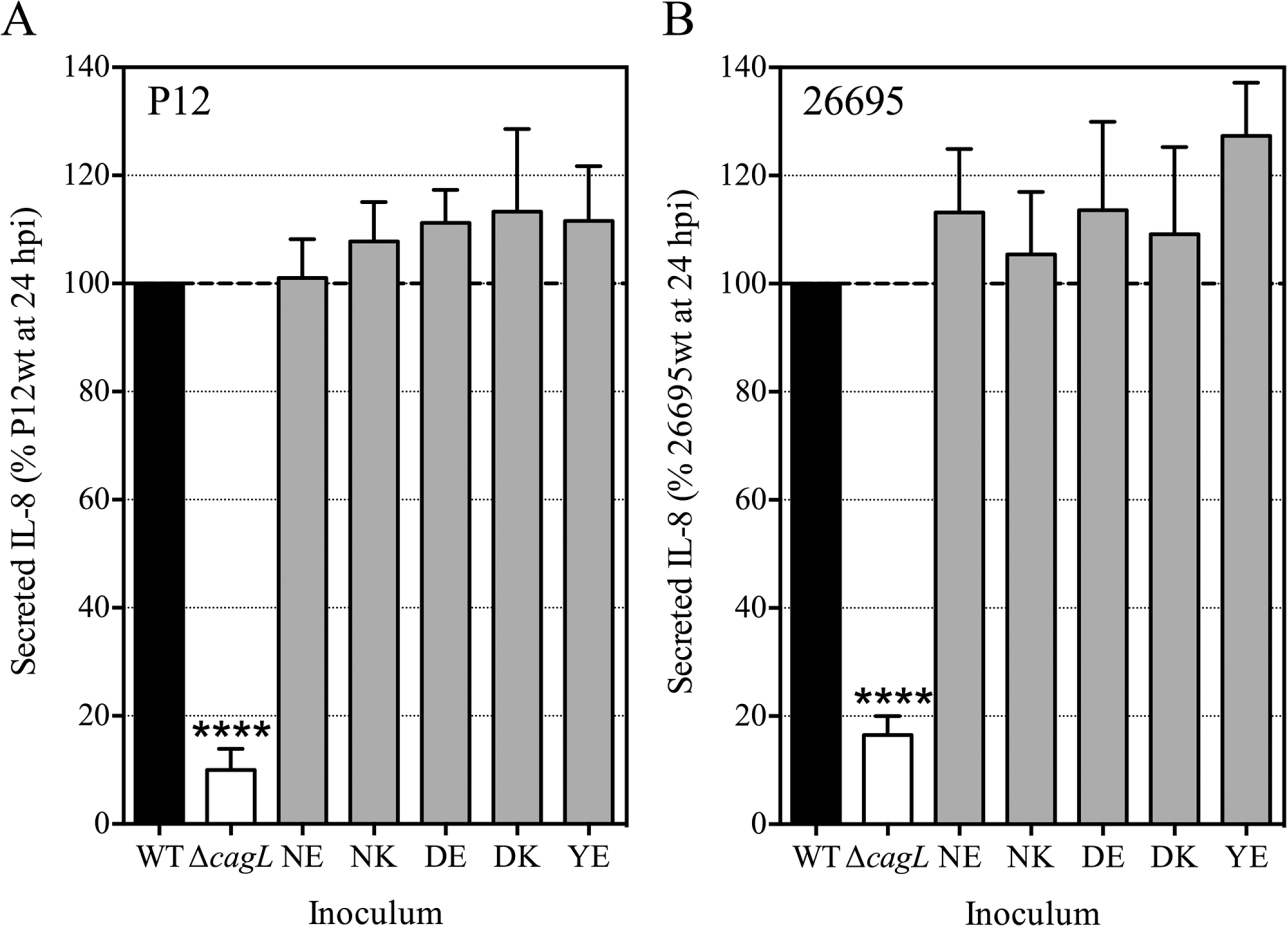
AGS cell IL-8 secretion induced by *H*. *pylori* isogenic CagL^58/59^ substitution mutants. (A) P12 isogenic *cagL* variant strains and (B) 26695 isogenic *cagL* variant strains. Spent culture medium was collected at 24 hours post-inoculation (hpi) with wild-type (WT) (P12 = D58K59; 26695 = N58E59), ∆*cagL* complemented with 26695wt-derived *cagL* (NE) or with 26695-derived *cagL* carrying substitutions at amino acids 58 and/or 59 (NK, DE, DK or YE). Samples were assayed for IL-8 levels by specific ELISA and data from individual experiments were standardized as a percentage of the level of IL-8 induced by isogenic WT strains; bars denote mean ± standard deviation (SD) from ≥3 independent experiments (each performed in duplicate). **** *P* <0.0001 against all other isogenic strains by one-way ANOVA with Tukey’s multiple comparisons test; all other comparisons not significant (*P* >0.05).

Akin to Tegtmeyer et al [11], reports that polymorphisms at CagL amino acid residues 58 and 59 could modulate CagL function with respect to host cell activation and CagA translocation [7, 10] also caught our attention. A previously generated P12∆*cagL* deletion mutant and a 26695 *cagL* knock-in plasmid construct [12] were used to produce variant *cagL* knock-in strains carrying the most common amino acid polymorphisms at residues 58 (N, D or Y) and 59 (E or K). We used these mutants to examine the contribution of amino acid diversity at these specific residues to *H*. *pylori*-mediated proinflammatory responses. The amino acid polymorphism combinations examined in this study were: D58K59 and Y58E59 which have both been linked with increased gastric cancer risk [7, 8]; and N58E59, N58K59 and D58E59 which have not shown a specific link to *H*. *pylori* disease progression [7–9]. These various P12 CagL amino acid substitution mutants were co-cultured with AGS gastric epithelial cells and the spent culture media was assayed for IL-8 secretion. Expression of *cagL* was confirmed by RT-PCR (S2 Fig). In contrast to Yeh et al [7], we did not observe significant differences in the level of IL-8 secretion in response to any of the P12 isogenic amino acid substitution strains compared to the wild-type *cagL* knock-in strain (Fig 1A). This was verified for duplicate independent clones at both 6 and 24 hpi (S3 Fig). Similar results were obtained using 26695 isogenic CagL variant strains (Fig 1B). The IL-8 secretion data suggested full functioning of the *cag* PAI type IV secretion system (T4SS) was not modulated solely by polymorphisms at residues 58 and/or 59.

**Fig 2:**
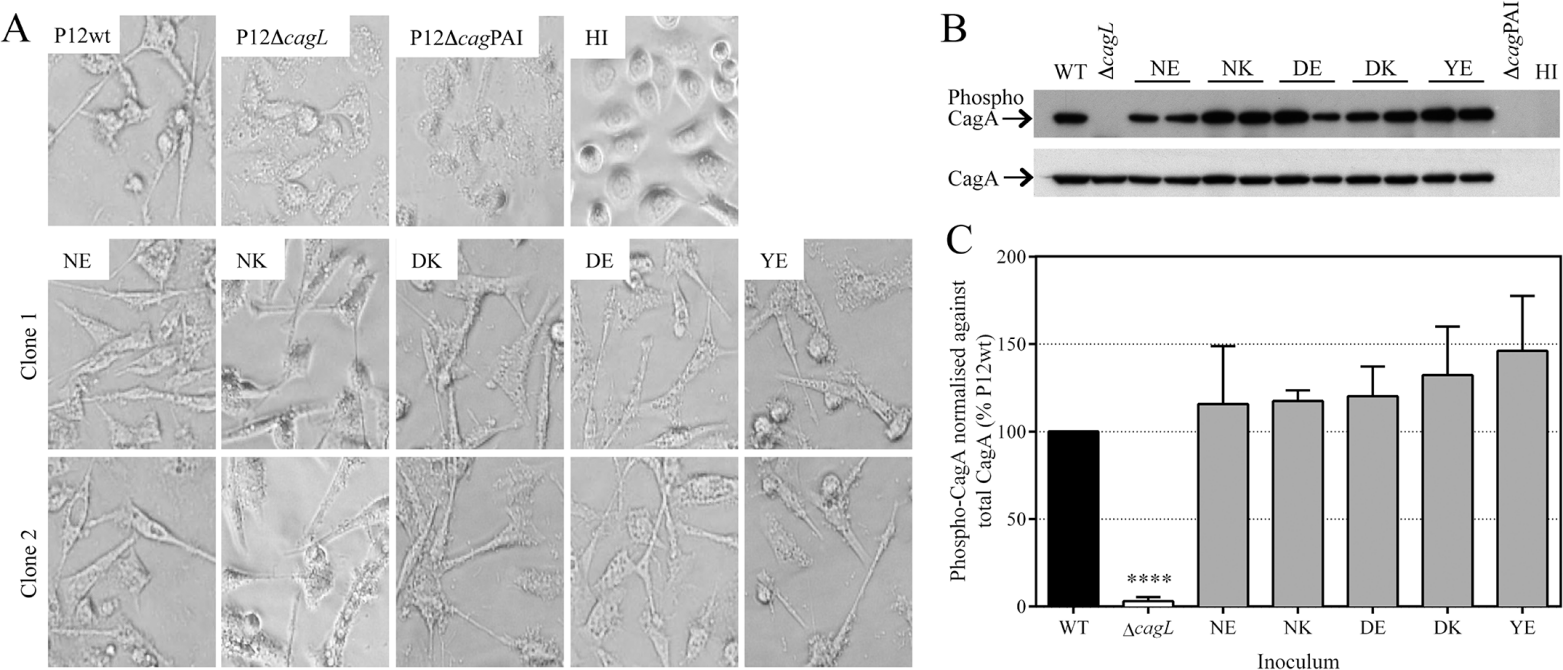
CagA translocation into AGS cells by CagL^58/59^ substitution mutants. (A) Hummingbird morphology of AGS cells in response to *H*. *pylori* P12 WT, P12∆*cagL* and the CagL^**58/59**^ substitution or knock-in variants, P12*cagL* ^**wt(NE)**^, P12*cagL* ^**NK**^, P12*cagL* ^**DE**^, P12*cagL* ^**DK**^, P12*cagL* ^**YE**^, at 8 hpi; images of duplicate independent clones (clones 1 and 2) shown. (B) Immunoblot analysis of phosphorylated CagA in AGS cell lysates harvested at 24 hpi with *H*. *pylori* strains P12 WT, P12∆*cagL*, and various P12 CagL^**58/59**^ substitution or knock-in variants, or sterile culture media (HI). (C) Densitometry data of 24 hpi immunoblot analysis; bars denote mean ± SD from 3 independent experiments; **** *P* <0.0001 against all other isogenic strains by one-way ANOVA with Tukey’s multiple comparisons test; all other comparisons not significant (*P* >0.05). See S5 Fig for entire blots.

Wild-type-like T4SS function of the various CagL 58/59 substitution mutants was further indicated by the development of hummingbird elongation of AGS cells in response to these strains that was indistinguishable from that induced by wild-type P12 or P12*cagL* ^WT^ strains (Fig 2A). We further examined their T4SS function by measuring CagA translocation in infected AGS cell-lysates by immunoblot analysis using phosphotyrosine- and CagA-specific antibodies. All P12 *cagL* knock-in variant strains showed efficient translocation of CagA into the gastric epithelial cells (Fig 2B) that was not significantly different to that observed with the wild-type complemented strain (Fig 2C). Similarly, wild-type-like T4SS functions were observed with CagL 58/59 substitution variants in strain 26695 (S4 Fig).

## Discussion

The recent reports by Yeh et al [10] and Tegtmeyer et al [11] showed that modification of CagL amino acid residues 58 and 59 modulated [10] or even ablated [11] function of the *cag* PAI-encoded type IV secretion system. Their findings suggested that these hypervariable residues of CagL were critical for *H*. *pylori* pathogenesis. Independently of these previous studies, we have observed that modification of amino acids at these residues alone was insufficient to modulate CagA translocation or *H*. *pylori*-mediated IL-8 secretion by gastric epithelial cells.

Our findings differ from those of Yeh et al who found that polymorphisms D58E59 and D58K59 were associated with significantly reduced IL-8 induction when introduced into CagL of the gastric cancer-associated *H*. *pylori* strain Hp1033 compared to the Y58E59 polymorphism carried by the isogenic wild-type [10]. Interestingly the Hp1033 D58K59, but not D58E59, variant also showed measurably reduced CagA translocation compared to Hp1033 Y58E59. Here we used well defined strains originally isolated from a gastritis patient (26695) and a duodenal ulcer patient (P12), into which we introduced polymorphisms that have either been suggested to be gastric cancer associated or non-disease associated [7–9]. In contrast to the observations of Yeh et al [10], our comparison of IL-8 responses and CagA translocation using isogenic Y58E59, D58K59 and D58E59 variants in addition to N58K59 and N58E59 variants in both P12 and 26695 backgrounds yielded no statistically significant difference between any of the polymorphisms. The natural amino acid sequence at residues 58–62 of CagL in *H*. *pylori* strain Hp1033 is YEIGK [10] whereas that in 26695 is NEMGE; thereby the CagL of these strains also differ at residues 60 and 62. In addition to these differences within the hypervariable motif, there are six additional amino acid polymorphisms between Hp1033 and 26695 of which four involve charged residues (S6 Fig). It is therefore possible that the differences between our observations and those of Yeh et al using Hp1033 could be due to variation in the CagL amino acid sequences of the different *H*. *pylori* strains used other than at residues 58/59. If this were the case, this would strongly argue that polymorphisms at CagL residues 58/59 do not influence *H*. *pylori* pathogenesis in isolation, but rather work in concert with other residues elsewhere in CagL to modulate CagL function. According to Rizzato et al [9], the *cagL* gene shows 74 nucleotide polymorphisms, including 31 non-synonymous polymorphisms. To date, only a subset of these 31 amino acid polymorphisms have been examined for cancer association: Yeh et al examined residues 58, 59, 122, 201, 210, 216, 221 and 234 [7]; Shukla et al examined residues 35, 58, 59, 60, 62 and 122 [8]; and Rizzato et al examined residues 35, 55, 58, 60 and 176 [9]. Of these, only polymorphisms at amino acid residues 55 [9], 58 [7–9] and 59 [7, 8] have been shown to correlate with host gastric cancer risk.

Apart from possible interstrain variation, differences in several technical aspects between this study and the Yeh et al study could have also contributed to the apparent outcome discrepancies. For example, although Yeh et al reported that substitution of Y58E59 by D58K59 in CagL significantly reduced the level of CagA translocation at pH 5.4, it is unclear whether such reduction was also observed at a neutral pH at which the experiments in both the Tegmeyer et al study [11] and this study were carried out. More importantly, no details have been provided in the Yeh et al report [10] on how the culture media pH was adjusted and maintained for the duration of the experiment or whether the variant strains had equivalent viability and/or growth rate under these stress conditions. These details are of particular importance for proper interpretation of data obtained from cells both maintained in an elevated CO_2_ environment and co-cultured with urease-producing, i.e. pH-modulating, *H*. *pylori*. Nor did Yeh et al rule out the presence of polar effects in their variant strains arising from their unique mutagenesis approach that did not exclude or screen for single-crossover recombination events, either during the original insertional mutagenesis or during the subsequent knock-in event.

Our findings based on the specific substitutions of only residues 58 and 59 also seemingly contradict those of Tegtmeyer et al who postulated that substitution of N58E59 by Y58E59 fully attenuated CagA translocation [11]. As pointed out by Tegtmeyer et al [11], the CagL Y58/E59 region of the gastric cancer-associated clinical isolates described in their study contains some additional amino acid variations at residue positions 60 and 62. However unlike in the Tegtmeyer et al study [11], we substituted only amino acid residues 58 and 59 but not residues 60 and 62, in order to specifically examine the role of amino acids 58 and 59 in CagL function and *H*. *pylori* pathogenesis. A plausible explanation for the apparent differences between our findings and those of Tegtmeyer et al [11] is that residues 60–62 might function in concert with residues 58 and 59 in influencing the structural integrity of CagL and its function in CagA translocation. It is possible that amino acid polymorphisms in this hinge region containing residues 58–62 could alter the length, conformation and/or flexibility of CagL, which may consequently alter accessibility of the RGD motif in α-helix 2 and/or the overall structure and functional activity of the protein. However given that this region of the CagL crystal structure is unresolved [6], it is not yet possible to accurately model the impact that the various different polymorphisms may have on overall CagL structure.

We postulate that the differences in the observed virulence effects of CagL 58/59 mutations between the Tegtmeyer et al [11] and Yeh et al [10] studies may also be a consequence of additional as yet unidentified amino acid residues in the gastric cancer strain Hp1033 used by Yeh et al being incompatible with the substitution of Y58E59 to D58K59. This may also be the case for the 26695 YEIGK mutant generated by Tegtmeyer et al. It is important to note that the *H*. *pylori* strain Hp1033 wild-type used in the Yeh et al mutagenesis study [10], which contains the amino acid sequence YEIGK at residues 58–62, is fully capable of CagA translocation. In contrast, the 26695 YEIGK mutant generated by Tegtmeyer et al [11], which contains exactly the same sequence in this region, is defective in CagA translocation. This alone is evidence that the CagL amino acid polymorphism Y58E59 *per se* is not an inherent downregulator of type IV secretion of CagA, and instead strongly argues that the functional effects of residues 58–62 in CagL might be context-dependent. As already noted, there are other differences in the CagL amino acid sequence between Hp1033 and 26995 (S6 Fig). These differences may alter the YEIGK context and hence CagL structure and function. Taken all together, it is highly likely that additional as yet unidentified amino acid residues elsewhere in CagL work in concert with residues 58/59 alone, or together with residues 60–62, in determining the overall activity of the protein. Analyses are underway in our laboratory to examine how these additional residues might contribute to the role of CagL in type IV secretion functions.

There is substantial evidence that CagL has multiple functions in addition to α_5_ß_1_-mediated T4SS function and CagA translocation, including modulating both gastric acid secretion [3, 16] via integrin α_v_ß_5_ [17] and epithelial cell morphology via both α_5_ß_1_ and α_v_ß_3_ [2]. However these additional functions remain to be examined in the context of CagL hypervariation. Dissecting at the molecular level why certain polymorphisms in the CagL hypervariable region may correlate epidemiologically with disease outcome will likely require analysis of these other functions of CagL. It also requires sequence and structural analyses of the hypervariable region in the context of the entire CagL open reading frame as suggested by the findings of this and various other recent studies [7–9]. To thoroughly understand the molecular basis for the correlation of polymorphisms in the CagL hypervariable region with disease outcome, future studies should also address at least two important weaknesses evident in the relevant epidemiological studies to date [7–9]. The first is that only a small subset of partial CagL sequences from just one of these three studies [8] has been submitted to a publically available database for verification of the results. The second is that one of the studies examined only partial CagL sequences, and yet reported that the hypervariable region was the sole region carrying polymorphisms correlating with disease [8]. Future studies should ensure that complete CagL sequences are verified thoroughly and made available in public databases.

The question of whether polymorphisms in CagL, or any other protein(s) of *H*. *pylori*, are associated with severe disease progression is an important one to address as it is likely to (i) open new avenues for a better and earlier prediction of gastric cancer susceptibility in *H*. *pylori*-infected individuals and (ii) provide crucial insights into the molecular basis of *H*. *pylori*-induced gastric carcinogenesis. In agreement with Tegtmeyer et al [11], we believe that progress in this area will require robust analyses of a significantly larger number of CagL sequences of *H*. *pylori* strains from patients with different disease outcomes. To ensure that the scientific community has complete confidence in such analyses and that the findings obtained will have maximal translational potential, it is important that future studies are performed in a more transparent, comprehensive and verifiable manner than those performed to date.

## Supporting Information

S1 Fig Sequence analysis of *cagL* variant mutants generated in this study. Sequencing chromatograms of (A) two independent clones (Clone 1 and Clone 2) of P12*cagL* ^DK^, P12*cagL* ^YE^, P12*cagL* ^DE^ and P12*cagL* ^NK^; (B) single clones of 26695*cagL* ^DK^, 26695*cagL* ^YE^, 26695*cagL* ^DE^ and 26695*cagL* ^NK^.
(PDF)

S2 Fig Reverse transcription PCR analysis of *cagL* transcription during co-culture with AGS cells. RNA purified from *H*. *pylori*-inoculated AGS cells at 3 hpi was used as a template for *cagL* PCR either following reverse transcription for cDNA production (RT) or without cDNA production (no RT). The *cagL*-specific amplicon was 320 bp; *cagL* expression was not observed in P12∆*cagL* mutant but was successfully restored in all P12 CagL substitution mutants.
(PDF)

S3 Fig AGS cell IL-8 secretion induced by independent replicate clones of *H*. *pylori* isogenic CagL^58/59^ substitution mutants. Spent culture medium was collected at 6 or 24 hours post-inoculation with P12 wild-type (WT; D58K59), P12∆*cagL*, P12∆*cagL* knocked-in with 26695wt-derived *cagL* (NE) or two independent replicate clones (denoted by Arabic numerals) of P12∆*cagL* knocked-in with 26695-derived *cagL* carrying substitutions at amino acids 58 and/or 59 (NK, DE, DK or YE). Samples were assayed for IL-8 levels by ELISA and data from individual experiments were standardized as a percentage of IL-8 secretion by isogenic WT strain at 24 hours post-inoculation to correct for interassay variability in absolute IL-8 levels, and allow pooling of data from multiple independent experiments for statistical analysis; bars denote mean ± SD from ≥3 independent experiments (each performed in duplicate). ** P <0.01, **** *P* <0.0001 against all other isogenic strains at same time-point by two-way ANOVA with Tukey’s multiple comparisons test; all other comparisons not significantly different (*P* >0.05).
(PDF)

S4 Fig T4SS activity of 26695 CagL variant strains. (A) Hummingbird morphology of AGS cells in response to *H*. *pylori* 26695 CagL^58/59^ substitution mutants at 8 hpi. (B) Immunoblot analysis of phosphorylated CagA in AGS cell lysates harvested at 24 hpi with *H*. *pylori* strains 26695 wt, 26695∆*cagL*, 26695*cagL* ^wt(NE)^, 26695*cagL* ^NK^, 26695*cagL* ^DE^, 26695*cagL* ^DK^, 26695*cagL* ^YE^, or sterile culture media (HI). Samples separated over a single SDS-PAGE gel, immunoblotted onto a single membrane and sequentially probed using anti-phosphotyrosine-specific monoclonal antibody PY99 followed by CagA-specific polyclonal antisera; samples have been reordered for visual ease (see S5 Fig for original blots).
(PDF)

S5 Fig Original phosphotyrosine (A and C) and CagA (B and D) immunoblots shown in Fig 2 (A and B) and S4 Fig (C and D). Refer to legends of Fig 2 and S4 Fig for experimental details.
(PDF)

S6 Fig Alignment of CagL amino acid sequences of *H*. *pylori* strains (A) Hp1033 versus 26695, and (B) P12 versus 26695. In addition to sequence diversity within the CagL hypervariable motif (CagLHM; boxed), *H*. *pylori* strains (A) Hp1033 and 26695 also differ at residues 35, 101, 122, 124, 210 and 216; and (B) P12 and 26695 differ at residues 114, 122, 134 and 200. RGD motif–solid line; RGD helper motif–broken line. Hp1033 sequence translated from published nucleotide sequence [20]; P12 and 26695 accession numbers ACJ07700.1 and NP_208335.1, respectively; CagL sequences were verified for P12 and 26695 stocks used in this study. Protein alignments performed using Emboss Needle pairwise sequence alignment server [21].
(PDF)

S1 Table *H*. *pylori* strains used in this study. References included in S1 Table: [12, 18, 19].
(PDF)

S2 Table Plasmids and primers used in this study. References included in S2 Table: [12].
(PDF)
