# Supplementary material for: Preservation of Helicobacter pylori CagA Translocation and Host Cell Proinflammatory Responses in the Face of CagL Hypervariability at Amino Acid Residues 58/59
Source: PLoS One. 2015 Jul 21;10(7):e0133531. doi: 10.1371/journal.pone.0133531 (PMC4509909; doi:10.1371/journal.pone.0133531)
Supplement: S1 Fig — Sequencing chromatograms of (A) two independent clones (Clone 1 and Clone 2) of P12cagL DK, P12cagL YE, P12cagL DE and P12cagL NK; (B) single clones of 26695cagL DK, 26695cagL YE, 26695cagL DE and 26695cagL NK. (PDF) [file pone.0133531.s001.pdf]

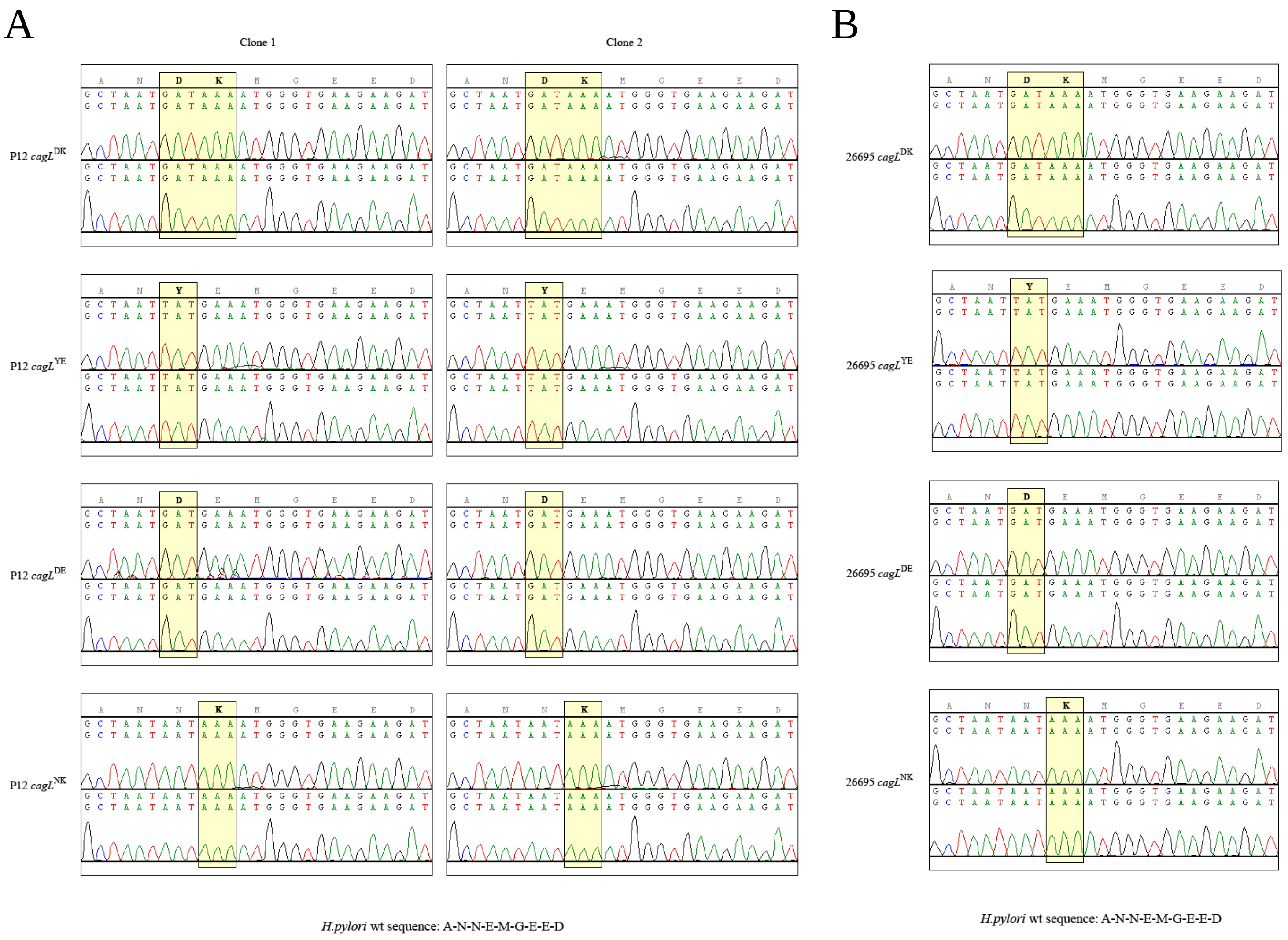


**S1 Figure. Sequence analysis of *cagL* variant mutants generated in this study.**

Sequencing chromatograms of (A) two independent clones (Clone 1 and Clone 2) of P12*cagL*$^{DK}$, P12*cagL*$^{YE}$, P12*cagL*$^{DE}$ and P12*cagL*$^{NK}$; (B) single clones of 26695*cagL*$^{DK}$, 26695*cagL*$^{YE}$, 26695*cagL*$^{DE}$ and 26695*cagL*$^{NK}$.
