# Supplementary material for: Preservation of Helicobacter pylori CagA Translocation and Host Cell Proinflammatory Responses in the Face of CagL Hypervariability at Amino Acid Residues 58/59
Source: PLoS One. 2015 Jul 21;10(7):e0133531. doi: 10.1371/journal.pone.0133531 (PMC4509909; doi:10.1371/journal.pone.0133531)
Supplement: S2 Fig — RNA purified from H. pylori-inoculated AGS cells at 3 hpi was used as a template for cagL PCR either following reverse transcription for cDNA production (RT) or without cDNA production (no RT). The cagL-specific amplicon was 320 bp; cagL expression was not observed in P12∆cagL mutant but was successfully restored in all P12 CagL substitution mutants. (PDF) [file pone.0133531.s002.pdf]

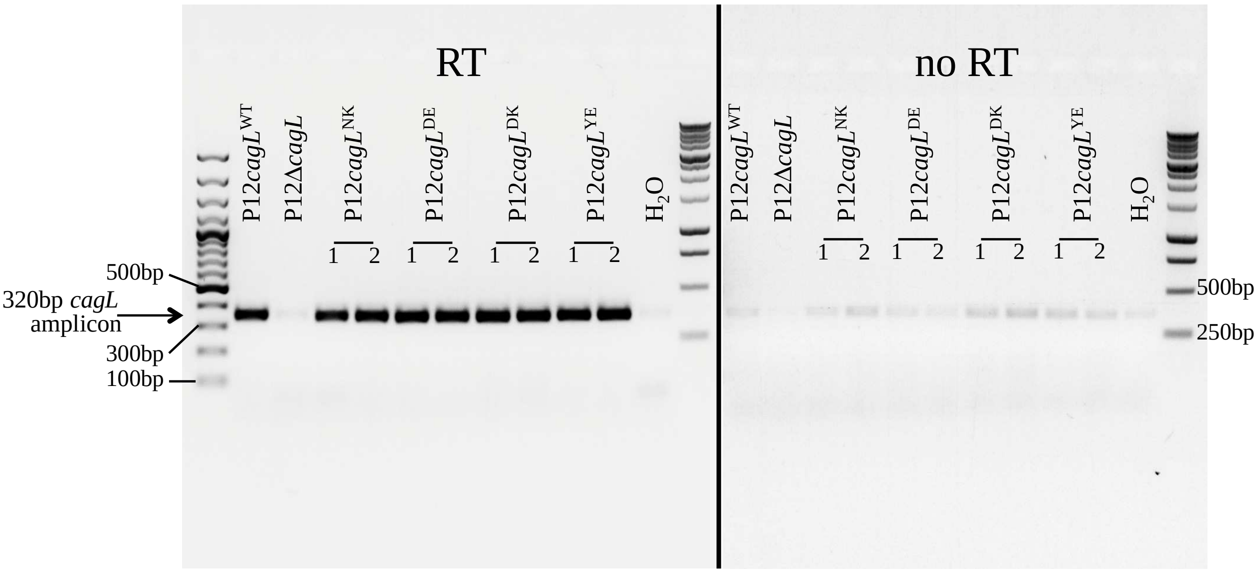

S2 Figure. Reverse transcription PCR analysis of *cagL* transcription during co-culture with AGS cells.

RNA purified from *H. pylori*-inoculated AGS cells at 3 hpi was used as a template for *cagL* PCR either following reverse transcription for cDNA production (RT) or without cDNA production (no RT). The *cagL*-specific amplicon was 320 bp; *cagL* expression was not observed in P12Δ*cagL* mutant but was successfully restored in all P12 CagL substitution mutants.
