# Supplementary material for: Preservation of Helicobacter pylori CagA Translocation and Host Cell Proinflammatory Responses in the Face of CagL Hypervariability at Amino Acid Residues 58/59
Source: PLoS One. 2015 Jul 21;10(7):e0133531. doi: 10.1371/journal.pone.0133531 (PMC4509909; doi:10.1371/journal.pone.0133531)
Supplement: S3 Fig — Spent culture medium was collected at 6 or 24 hours post-inoculation with P12 wild-type (WT; D58K59), P12∆cagL, P12∆cagL knocked-in with 26695wt-derived cagL (NE) or two independent replicate clones (denoted by Arabic numerals) of P12∆cagL knocked-in with 26695-derived cagL carrying substitutions at amino acids 58 and/or 59 (NK, DE, DK or YE). Samples were assayed for IL-8 levels by ELISA and data from individual experiments were standardized as a percentage of IL-8 secretion by isogenic WT strain at 24 hours post-inoculation to correct for interassay variability in absolute IL-8 levels, and allow pooling of data from multiple independent experiments for statistical analysis; bars denote mean ± SD from ≥3 independent experiments (each performed in duplicate). ** P <0.01, **** P <0.0001 against all other isogenic strains at same time-point by two-way ANOVA with Tukey’s multiple comparisons test; all other comparisons not significantly different (P >0.05). (PDF) [file pone.0133531.s003.pdf]

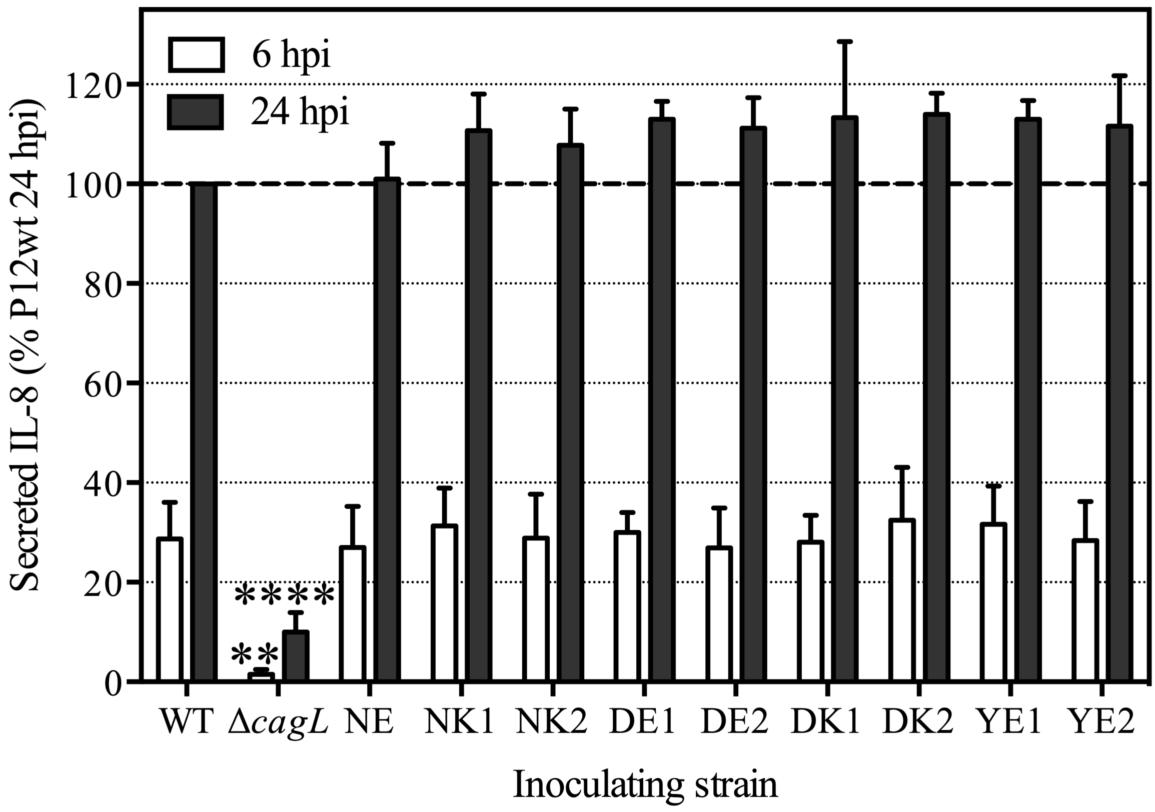

S3 Figure. AGS cell IL-8 secretion induced by independent replicate clones of *H. pylori* isogenic $CagL^{58/59}$ substitution mutants.

Spent culture medium was collected at 6 or 24 hours post-inoculation with P12 wild-type (WT; D58K59), P12Δ*cagL*, P12Δ*cagL* knocked-in with 26695wt-derived *cagL* (NE) or two independent replicate clones (denoted by Arabic numerals) of P12Δ*cagL* knocked-in with 26695-derived *cagL* carrying substitutions at amino acids 58 and/or 59 (NK, DE, DK or YE). Samples were assayed for IL-8 levels by ELISA and data from individual experiments were standardized as a percentage of IL-8 secretion by isogenic WT strain at 24 hours post-inoculation to correct for interassay variability in absolute IL-8 levels, and allow pooling of data from multiple independent experiments for statistical analysis; bars denote mean ± SD from ≥3 independent experiments (each performed in duplicate). ** P <0.01, **** *P* <0.0001 against all other isogenic strains at same time-point by two-way ANOVA with Tukey's multiple comparisons test; all other comparisons not significantly different (*P* >0.05).
