# Supplementary material for: Preservation of Helicobacter pylori CagA Translocation and Host Cell Proinflammatory Responses in the Face of CagL Hypervariability at Amino Acid Residues 58/59
Source: PLoS One. 2015 Jul 21;10(7):e0133531. doi: 10.1371/journal.pone.0133531 (PMC4509909; doi:10.1371/journal.pone.0133531)
Supplement: S4 Fig — (A) Hummingbird morphology of AGS cells in response to H. pylori 26695 CagL58/59 substitution mutants at 8 hpi. (B) Immunoblot analysis of phosphorylated CagA in AGS cell lysates harvested at 24 hpi with H. pylori strains 26695 wt, 26695∆cagL, 26695cagL wt(NE), 26695cagL NK, 26695cagL DE, 26695cagL DK, 26695cagL YE, or sterile culture media (HI). Samples separated over a single SDS-PAGE gel, immunoblotted onto a single membrane and sequentially probed using anti-phosphotyrosine-specific monoclonal antibody PY99 followed by CagA-specific polyclonal antisera; samples have been reordered for visual ease (see S5 Fig for original blots). (PDF) [file pone.0133531.s004.pdf]

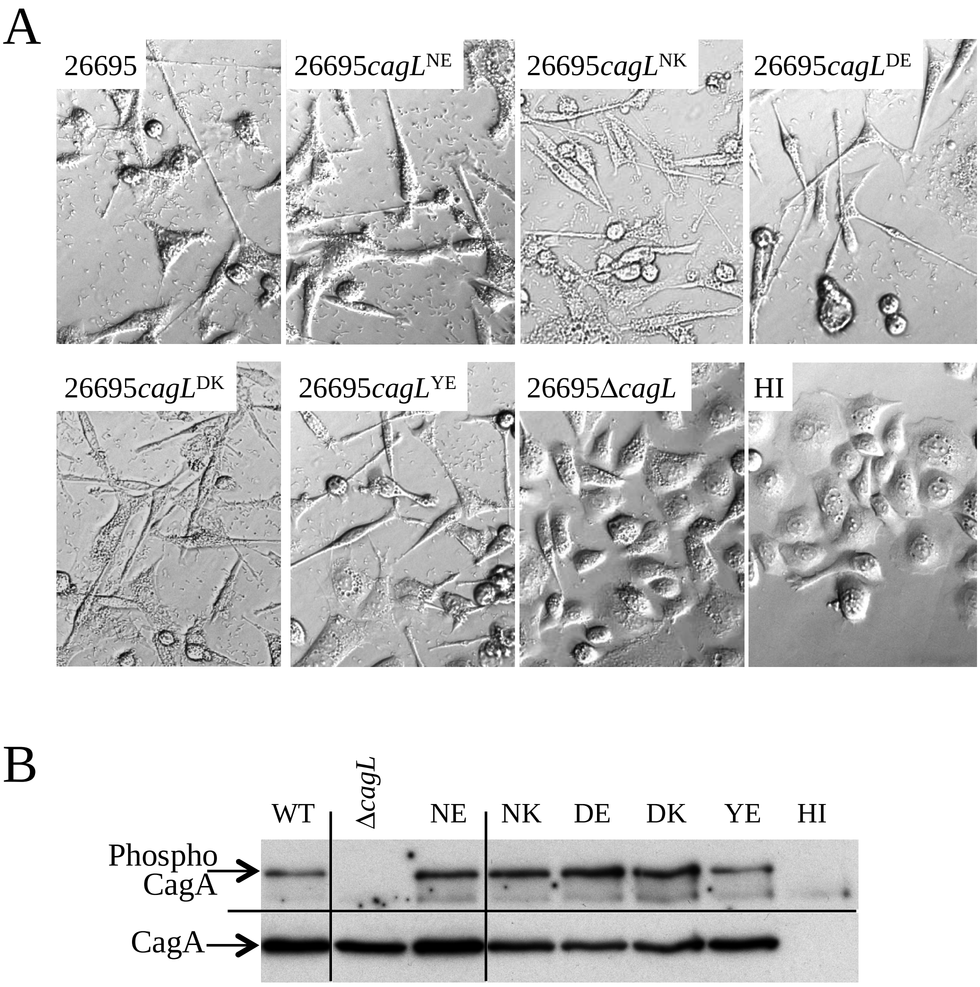

S4 Figure. T4SS activity of 26695 CagL variant strains.

(A) Hummingbird morphology of AGS cells in response to *H. pylori* 26695 CagL$^{58/59}$ substitution mutants at 8 hpi. (B) Immunoblot analysis of phosphorylated CagA in AGS cell lysates harvested at 24 hpi with *H. pylori* strains 26695 wt, 26695Δ*cagL*, 26695*cagL*$^{wt(NE)}$, 26695*cagL*^{NK}, 26695*cagL*^{DE}, 26695*cagL*^{DK}, 26695*cagL*^{YE}, or sterile culture media (HI). Samples separated over a single SDS-PAGE gel, immunoblotted onto a single membrane and sequentially probed using anti-phosphotyrosine-specific monoclonal antibody PY99 followed by CagA-specific polyclonal antisera; lanes have been digitally reordered for visual ease (see S5 Figure for original blots).
