# Supplementary material for: Preservation of Helicobacter pylori CagA Translocation and Host Cell Proinflammatory Responses in the Face of CagL Hypervariability at Amino Acid Residues 58/59
Source: PLoS One. 2015 Jul 21;10(7):e0133531. doi: 10.1371/journal.pone.0133531 (PMC4509909; doi:10.1371/journal.pone.0133531)
Supplement: S5 Fig — Refer to legends of Fig 2 and S4 Fig for experimental details. (PDF) [file pone.0133531.s005.pdf]

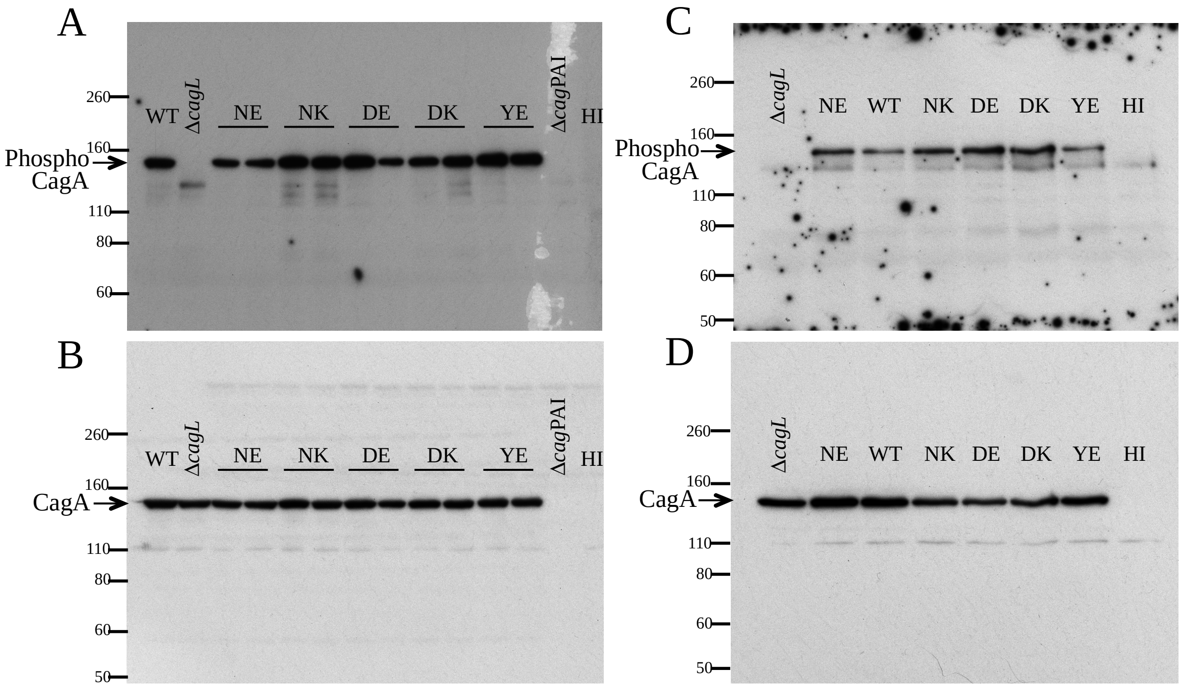

S5 Figure. Original phosphotyrosine (A and C) and CagA immunoblots (B and D) shown in Fig. 2 (A and B) and S4 Figure (C and D).

Refer to legends of Fig. 2 and S4 Figure for experimental details.
