# Supplementary material for: Preservation of Helicobacter pylori CagA Translocation and Host Cell Proinflammatory Responses in the Face of CagL Hypervariability at Amino Acid Residues 58/59
Source: PLoS One. 2015 Jul 21;10(7):e0133531. doi: 10.1371/journal.pone.0133531 (PMC4509909; doi:10.1371/journal.pone.0133531)
Supplement: S6 Fig — In addition to sequence diversity within the CagL hypervariable motif (CagLHM; boxed), H. pylori strains (A) Hp1033 and 26695 also differ at residues 35, 101, 122, 124, 210 and 216; and (B) P12 and 26695 differ at residues 114, 122, 134 and 200. RGD motif–solid line; RGD helper motif–broken line. Hp1033 sequence translated from published nucleotide sequence [20]; P12 and 26695 accession numbers ACJ07700.1 and NP_208335.1, respectively; CagL sequences were verified for P12 and 26695 stocks used in this study. Protein alignments performed using Emboss Needle pairwise sequence alignment server [21]. (PDF) [file pone.0133531.s006.pdf]

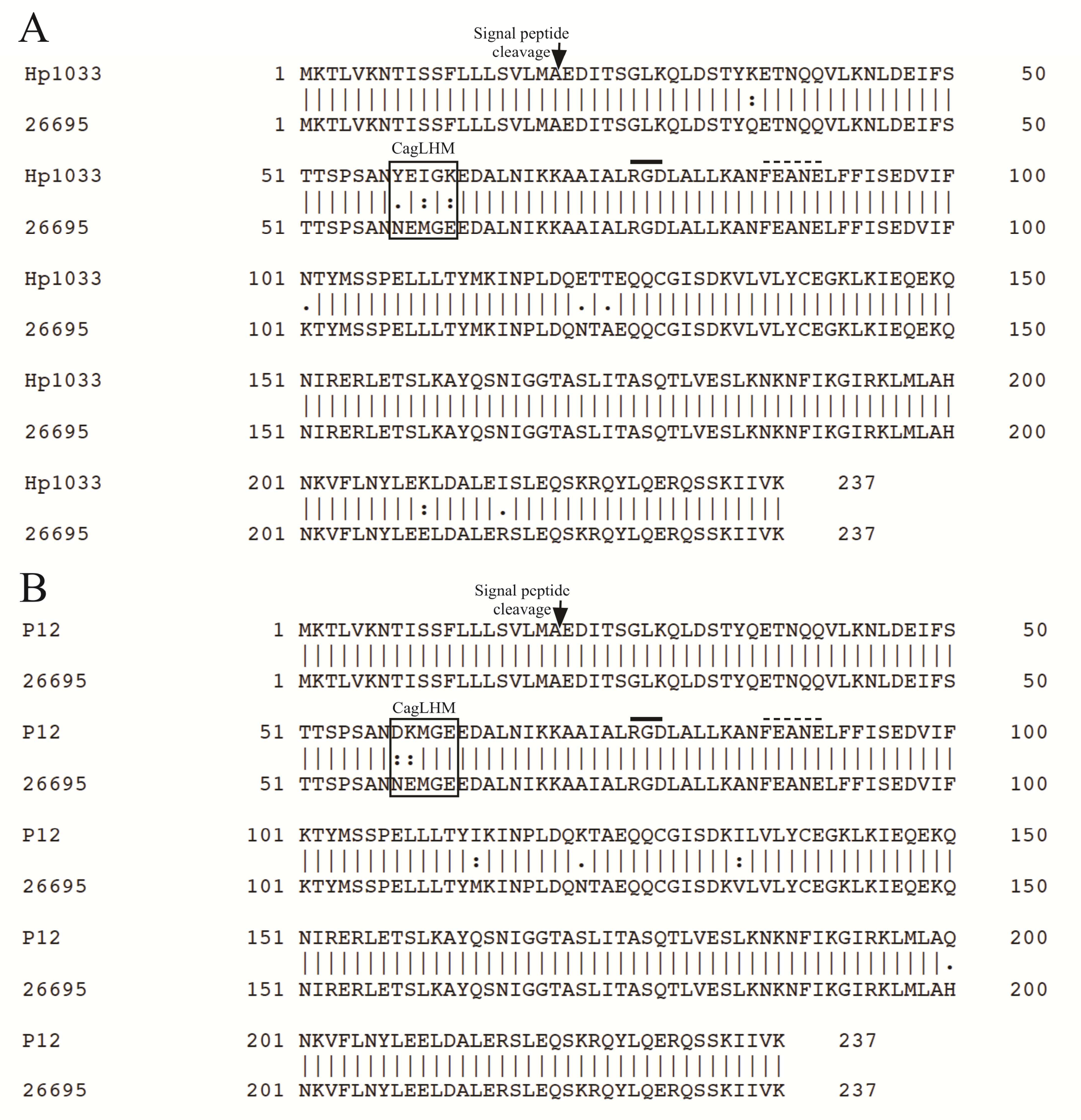

S6 Figure. Alignment of CagL amino acid sequences of *H. pylori* strains (A) Hp1033 versus 26695, and (B) P12 versus 26695.

In addition to sequence diversity within the CagL hypervariable motif (CagLHM; boxed), *H. pylori* strains (A) Hp1033 and 26695 also differ at residues 35, 101, 122, 124, 210 and 216; and (B) P12 and 26695 differ at residues 114, 122, 134 and 200. RGD motif – solid line; RGD helper motif – broken line. Hp1033 sequence translated from published nucleotide sequence [20]; P12 and 26695 accession numbers ACJ07700.1 and NP_208335.1, respectively; CagL sequences were verified for P12 and 26695 stocks used in this study. Protein alignments performed using Emboss Needle pairwise sequence alignment server [21].
