# Supplementary material for: Preservation of Helicobacter pylori CagA Translocation and Host Cell Proinflammatory Responses in the Face of CagL Hypervariability at Amino Acid Residues 58/59
Source: PLoS One. 2015 Jul 21;10(7):e0133531. doi: 10.1371/journal.pone.0133531 (PMC4509909; doi:10.1371/journal.pone.0133531)
Supplement: S1 Table — References included in S1 Table: [12, 18, 19]. (PDF) [file pone.0133531.s007.pdf]

S1 Table. *H. pylori* strains used in this study

Bacterial Strain	Genotype	References
P12	Wild-type	[18]
26695	Wild-type	[19]
P12Δ*cagL*	Complete *cagL* (HPP12_0546) deletion; Km^R	[12]
P12*cagL*^{NE}	P12Δ*cagL* knocked-in with *cagL* from p26695*cagL*^{WT}::*cat* (HP0539); $Cm^{R;}$ previously published as P12*cagL*^{WT}	[12]
P12*cagL*^{NK}	P12Δ*cagL* knocked-in with *cagL* from p26695*cagL*^{NK}::*cat*; Cm^R	This study
P12*cagL*^{DE}	P12Δ*cagL* knocked-in with *cagL* from p26695*cagL*^{DE}::*cat*; Cm^R	This study
P12*cagL*^{DK}	P12Δ*cagL* knocked-in with *cagL* from p26695*cagL*^{DK}::*cat*; Cm^R	This study
P12*cagL*^{YE}	P12Δ*cagL* knocked-in with *cagL* from p26695*cagL*^{YE}::*cat*; Cm^R	This study
26695Δ*cagL*	Complete *cagL* (HP0539) deletion; Km^R	This study
26695*cagL*^{NE}	26695Δ*cagL* knocked-in with *cagL* from p26695*cagL*^{WT}::*cat* (HP0539); Cm^R	This study
26695*cagL*^{NK}	26695Δ*cagL* knocked-in with *cagL* from p26695*cagL*^{NK}::*cat*; Cm^R	This study
26695*cagL*^{DE}	26695Δ*cagL* knocked-in with *cagL* from p26695*cagL*^{DE}::*cat*; Cm^R	This study
26695*cagL*^{DK}	26695Δ*cagL* knocked-in with *cagL* from p26695*cagL*^{DK}::*cat*; Cm^R	This study
26695*cagL*^{YE}	26695Δ*cagL* knocked-in with cagL from p26695*cagL*^{YE}::*cat*; Cm^R	This study
