# Supplementary material for: Preservation of Helicobacter pylori CagA Translocation and Host Cell Proinflammatory Responses in the Face of CagL Hypervariability at Amino Acid Residues 58/59
Source: PLoS One. 2015 Jul 21;10(7):e0133531. doi: 10.1371/journal.pone.0133531 (PMC4509909; doi:10.1371/journal.pone.0133531)
Supplement: S2 Table — References included in S2 Table: [12]. (PDF) [file pone.0133531.s008.pdf]

S2 Table. Plasmids and primers used in this study.

Plasmid	Genotype	References
p26695*cagL*^{WT}::*cat*	pGEM-T easy containing *cagL* plus *cagI* and *cagN* flanking regions (26695 genome nts 570713-571881)	[12]
p26695*cagL*^{NK}::*cat*	E59 to K59 substitution of p26695*cagL*^{WT}	This study
p26695*cagL*^{DE}::*cat*	N58 to D58 substitution of p26695*cagL*^{WT}	This study
p26695*cagL*^{DK}::*cat*	N58 to K58 & E59 to K59 substitutions of p26695*cagL*^{WT}	This study
p26695*cagL*^{YE}::*cat*	N58 to Y58 substitution of p26695*cagL*^{WT}	This study

Primer name	Sequence (5’ –> 3’)
R-CagL58/59	ATTAGCACTAGGGCTAGTGGTTG
F-CagLN58/K59	AATAAAATGGGTGAAGAAGATGCTC
F-CagLD58/E59	GATGAAATGGGTGAAGAAGATGCTC
F-CagLY58/E59	TATGAAATGGGTGAAGAAGATGCTC
F-CagLD58/K59	GATAAAATGGGTGAAGAAGATGCTC
